# Does the Temporal Asymmetry of Short-Term Heart Rate Variability Change during Regular Walking? A Pilot Study of Healthy Young Subjects

**DOI:** 10.1155/2018/3543048

**Published:** 2018-04-30

**Authors:** Xinpei Wang, Chang Yan, Bo Shi, Changchun Liu, Chandan Karmakar, Peng Li

**Affiliations:** ^1^School of Control Science and Engineering, Shandong University, Jinan, Shandong 250061, China; ^2^Department of Medical Imaging, Bengbu Medical College, Bengbu, Anhui 233030, China; ^3^School of Information Technology, Deakin University, Burwood, VIC 3125, Australia

## Abstract

The acceleration and deceleration patterns in heartbeat fluctuations distribute asymmetrically, which is known as heart rate asymmetry (HRA). It is hypothesized that HRA reflects the balancing regulation of the sympathetic and parasympathetic nervous systems. This study was designed to examine whether altered autonomic balance during exercise can lead to HRA changes. Sixteen healthy college students were enrolled, and each student undertook two 5-min ECG measurements: one in a resting seated position and another while walking on a treadmill at a regular speed of 5 km/h. The two measurements were conducted in a randomized order, and a 30-min rest was required between them. RR interval time series were extracted from the 5-min ECG data, and HRA (short-term) was estimated using four established metrics, that is, Porta's index (PI), Guzik's index (GI), slope index (SI), and area index (AI), from both raw RR interval time series and the time series after wavelet detrending that removes the low-frequency component of <~0.03 Hz. Our pilot data showed a reduced PI but unchanged GI, SI, and AI during walking compared to resting seated position based on the raw data. Based on the wavelet-detrended data, reduced PI, SI, and AI were observed while GI still showed no significant changes. The reduced PI during walking based on both raw and detrended data which suggests less short-term HRA may underline the belief that vagal tone is withdrawn during low-intensity exercise. GI may not be sensitive to short-term HRA. The reduced SI and AI based on detrended data suggest that they may capture both short- and long-term HRA features and that the expected change in short-term HRA is amplified after removing the trend that is supposed to link to long-term component. Further studies with more subjects and longer measurements are warranted to validate our observations and to examine these additional hypotheses.

## 1. Introduction

Under healthy physiological conditions, the human heart does not beat at a constant frequency; instead, heart rate changes all the time. This phenomenon has been recognized as heart rate variability (HRV) [1, 2]. For a given observation scale, the acceleration and deceleration patterns in beat-to-beat heart rate fluctuations distribute asymmetrically rather than contribute equally to HRV [3–7]. This suggests that the underlying heart rate control mechanisms—the regulation of sympathetic and parasympathetic nervous systems—are physiologically disproportionate over fixed temporal scales [8–12]. This asymmetry of acceleration and deceleration runs is defined as heart rate asymmetry (HRA).

In clinical settings, the electrocardiograms (ECGs) are commonly collected under well-controlled conditions such as resting supine or seated position and within a short time range (e.g., 5 min or shorter). Increasing attention nowadays has been drawn to the ambulatory ECG monitoring [13], which facilitates the tracking of heart rate and HRV with activities of free living, such as walking and exercise [14]. Long-term ambulatory measurement also assists to examine whether and how HRV properties respond to these daily activities [15]. Besides, daily activities may also evoke changes that may mask the effects of interest, for example, the changes that are related to alterations of health status or different times of the day. Thus, the examination of the changes of different HRV measures with daily activities may help better understand the variation profile of these measures, providing opportunities to comprehend the knowledge of how these novel properties respond to the changing physiological conditions that eventually should be of great help to develop sensitive and specific makers for cardiovascular diseases. With such a motivation, this study focused on elucidating whether and how the daily activities alter HRA.

The high-frequency power of HRV is accepted to be related to the parasympathetic tone while HRA has shown to be positively correlated with the high-frequency power [16], offering the link between HRA and parasympathetic activity. This link has further been strengthened by the observations that parasympathetic block leads to less prevalence of HRA [16] and that the deceleration patterns have a larger contribution to short-term HRA than acceleration patterns [9, 12]. Based on these existing results, we expect to see a significantly reduced short-term HRA level during low-intensity daily exercises that are assumed to be accompanied with the withdrawal of parasympathetic modulation [17]. In the current study, we applied treadmill-based regular walking protocol to imitate daily exercises in laboratory. To examine the within-subject changes, each participant undertook a walking protocol and a rest protocol. During each protocol, ECG data were collected continuously for 5 minutes. The next section explains in detail the subjects, experimental protocols, and analysis methods. Experimental results are summarized in the Results, followed by discussions in the Discussions.

## 2. Methods

### 2.1. Subjects

Subjects include 16 college students (4 females, 12 males; age: 20.1 ± 0.6 years [mean ± standard deviation]) with their physical and mental health status confirmed by questionnaire on the history of cardiovascular diseases, diabetes, depression, and neurological disorders. No subject has been taking any medications that have known effects on ANS within two weeks before participation. Adequate sleep during the night before coming to the laboratory, as well as avoidance of vigorous exercises during the test day and the day before, was requested. Written informed consent was obtained from all subjects. The study was approved by the Ethics Committee in Clinical Study of Bengbu Medical College.

### 2.2. Protocols

For each subject, ECG was recorded twice in random order with the subject seating on a chair or walking on a treadmill (ZR11, Reebok, Canton, MA, USA) at a speed of 5 km/h. Both ECGs last for 5 min and a 30-min rest was scheduled between the two measurements. Holter monitors (DiCare-mlCP, Dimetek Digital Medical Tech., Ltd., Shenzhen, China) were used to collect ECG data. The sampling frequency was 200 Hz, and standard unipolar chest lead V5 was applied. All the measurements were undertaken in a quiet, temperature-controlled (23 ± 1 degree Celsius) room.

### 2.3. Construction of HRV Time Series

ECGs were first subjected to a visual quality inspection assisted by a self-designed MATLAB program with user interface, which confirmed that all recordings were with high signal qualities. A template-matching process was then applied to extract the R peaks [18] followed by a second-round visual inspection for the correction of misidentified peaks and ectopic beats using the same MATLAB program. During this visual inspection, false positive detection was removed while false negatives were filled with the actual location of R peaks read manually from the program. We confirmed that no ectopic beats occurred in those data. HRV time series were finally constructed by the consecutive R-R intervals.

### 2.4. HRA Metrics

The following four well-established metrics derived from the Poincaré plot were calculated.

#### 2.4.1. Porta's Index (PI)

Conceptually, PI renders symmetry when the numbers of points in the two regions in Poincaré plot separated by the line of identity (LI) are the same and renders asymmetry if they differ [19]. Different levels of asymmetry can be estimated by how much the numbers differ. Thus, PI can be calculated by

(1) $$\begin{array}{c} \text{PI}=\frac{a}{m}\times 100, \end{array}$$

wherein *a* is the number of points above LI and *m* the total number of points (points on LI excluded).

#### 2.4.2. Guzik's Index (GI)

GI uses the distances between points and LI as a measure to assess whether the contributions of points in the two different regions in Poincaré plot are equal or not [20]. Specifically,

(2) $$\begin{array}{c} \text{GI}=\frac{\sum_{i=1}^{a}D_{i}}{\sum_{i=1}^{m}D_{i}}\times 100, \end{array}$$

wherein *D*_*i*_ is the Euclidian distance of point *i* to LI. For the RR interval time series, the Poincaré plot is actually to plot the current RR interval versus its subsequent interval. Thus, $D_{i}=\left|{\text{RR}}_{i+1}-{\text{RR}}_{i}\right|/\sqrt{2}$.

#### 2.4.3. Slope Index (SI)

The average phase angles of points in the two different regions in Poincaré plot are calculated and used to assess the asymmetry [21]. Specifically,

(3) $$\begin{array}{c} \text{SI}=\frac{\sum_{i=1}^{a}\left|R\theta_{i}\right|}{\sum_{i=1}^{m}\left|R\theta_{i}\right|}\times 100, \end{array}$$

wherein *Rθ*_*i*_ = *π*/4 − *θ*_*i*_. *θ*_*i*_ = atan⁡(RR_*i*+1_/RR_*i*_) is the phase angle of point *i* and *π*/4 is the point angle of LI, that is, atan⁡(1).

#### 2.4.4. Area Index (AI)

The average areas of sectors formed by the points and LI are calculated and used to assess the asymmetry [22]. Specifically,

(4) $$\begin{array}{c} \text{AI}=\frac{\sum_{i=1}^{a}S_{i}}{\sum_{i=1}^{m}S_{i}}\times 100, \end{array}$$

wherein *S*_*i*_ = 1/2 × *Rθ*_*i*_ × *r*^2^ is the area of the sector formed by point *i* and LI. *r* is the radius of the sector.

### 2.5. HRA Analysis of Short-Term HRV

**Figure 1:**
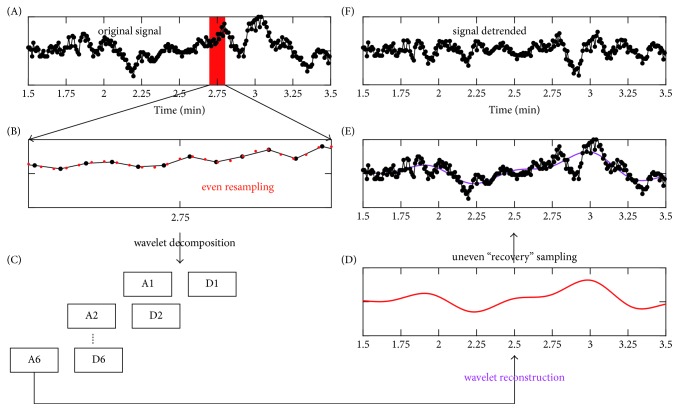
Wavelet-based nonstationary trend removal procedure. (A) The original RR interval time series. (B) The RR interval time series after the even resampling. In order to clearly demonstrate the even resampling time points (red dots) and the original time points (black dots), a segment of data from (A) is zoomed in and shown in this panel. (C) The 6-level wavelet decomposition. (D) Trend with even sampling points is obtained from wavelet reconstruction of the approximate coefficients on the 6th level. (E) The actual nonstationary trend (purple) is obtained from the uneven recovering sampling from the trend component in panel (D). (F) The detrended RR interval time series is obtained by subtracting the actual nonstationary trend from the original RR interval time series.

The four HRA metrics were performed on HRV data collected under both conditions. The asymmetry level was further defined as the deviation of a specific HRA metric from its level for completely symmetrical data, that is, |*x* − 50| (*x* denotes an HRA metric), and was denoted as ΔPI, ΔGI, ΔSI, and ΔAI, respectively. Besides, to explore the potential effect of nonstationary trend, wavelet detrending was performed and the above four asymmetrical indices were recalculated using the detrended data. To perform the wavelet detrending, raw HRV data were first evenly resampled to 4 Hz by spline interpolation. A 6-level wavelet decomposition using the coif5 wavelet was then conducted. The approximation coefficients on the 6th level were reconstructed to the original scale and were nonevenly “recovered” by spline interpolation which resulted in the trend that would be subtracted. The 6-level decomposition was used so that the frequency band of the trend would be less than ~0.03 Hz.  Figure 1 intuitively demonstrates this wavelet detrending procedure.

### 2.6. Statistical Analysis

The Shapiro–Wilk *W* test suggested nonnormal distribution of all the HRA results. Therefore, the Wilcoxon signed-rank test of each pair was used to examine the within-subject differences under the two measurement conditions. In addition, Cohen's *d* static was calculated for statistically significant observations to examine the effect size of the corresponding metric. A medium effect size was considered if *d* ≥ 0.5 and large if *d* ≥ 0.8 [23]. As secondary analysis, we also performed the Wilcoxon signed-rank tests by restricting to male subjects (*N* = 12) only. We did not perform these tests separately on females as we only had 4 females. All the statistical analyses were performed using the JMP software (Pro 13, SAS Institute, Cary, NC, USA).

## 3. Results

**Figure 2:**
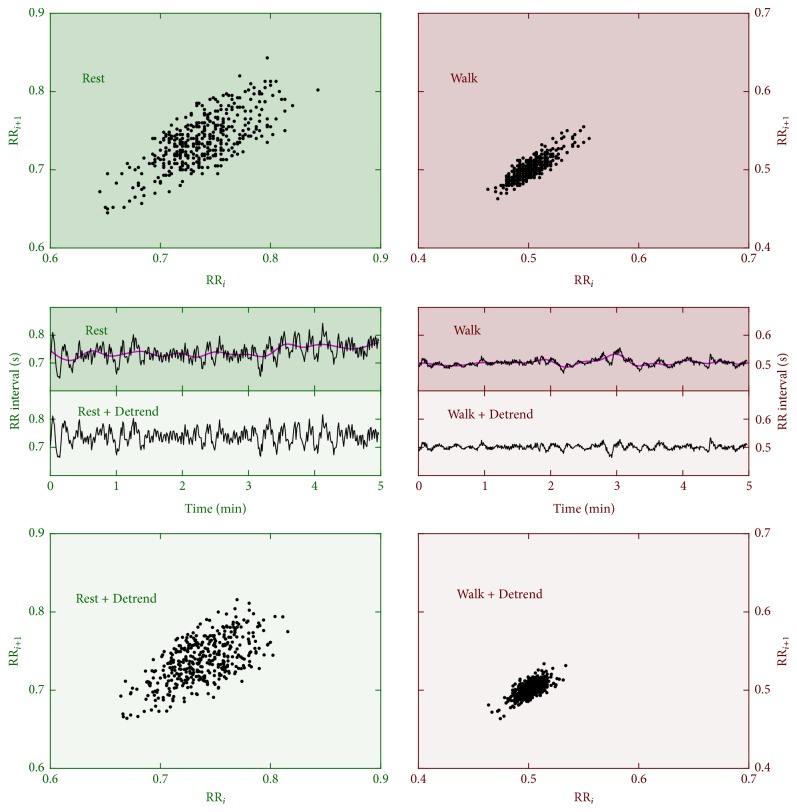
Exemplary RR interval time series (middle four panels) and the corresponding Poincaré plots (upper two and lower two panels). Left: data during seated position, right: data during walking. Data after nonstationary trend removal are shown with light-shaded background colors on lower four panels with “+Detrend” legend.

A typical RR interval time series for resting seated position and the corresponding RR interval time series from the same subject during walking are shown in Figure 2. Overall, the RR intervals become shorter (i.e., heart beats faster) during walking, such that the points distribute more compactly on the Poincaré plot than those during rest if the same scale is used. The Poincaré plots also become more compact after nonstationary trend removal, which is expected because of the effect of detrending on long-term HRA.

### 3.1. Asymmetry Based on Raw HRV Time Series

**Figure 3:**
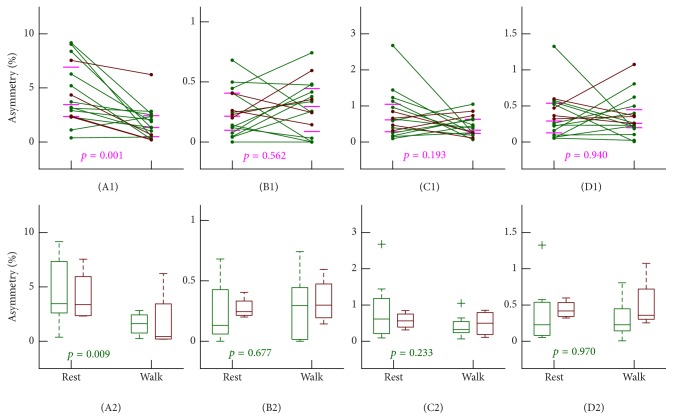
The asymmetries of short-term heart rate variability without detrending. In order to show the changes, results from the same individual were connected by lines. Horizontal bars indicate the median and [1st, 3rd] quartiles. *p* values were from Wilcoxon signed-rank test of each pair. ((A1) and (A2)) ΔPI; ((B1) and (B2)) ΔGI; ((C1) and (C2)) ΔSI; ((D1) and (D2)) ΔAI.* Rest*: results under resting seated position;* Walk*: results during regular walking. 14 individuals show reduction from* Rest* to* Walk* in (A1). Results from males and from females are marked in different colors (male: green; female: brown). Lower panels summarize the box plots (that show from top to bottom the max, 3rd quartile, median, 1st quartile, and min) for males and females, separately. Outliers, if there are any, are marked by “+.”

HRV data collected under both conditions displayed asymmetry as assessed by the four HRA metrics (all four* p*'s < 0.001 under both conditions as revealed by Wilcoxon signed-rank test of each measure versus symmetrical level; i.e., index = 0). Compared to the resting seated position, a significant reduction of HRA during walking was observed by PI (Wilcoxon signed-rank test of each pair: *p* = 0.001; Cohen's* d* = 1.0; out of the 16 subjects, 14 including all the four females showed reduction; Figure 3(A1)). No significant HRA changes during walking were suggested by the remaining three metrics (all* p*'s > 0.1; Figures 3(B1)–3(D1)). The results persisted when restricting the Wilcoxon signed-rank tests to male subjects only (Figures 3(A2)–3(D2)).

### 3.2. Asymmetry Based on Detrended HRV Time Series

**Figure 4:**
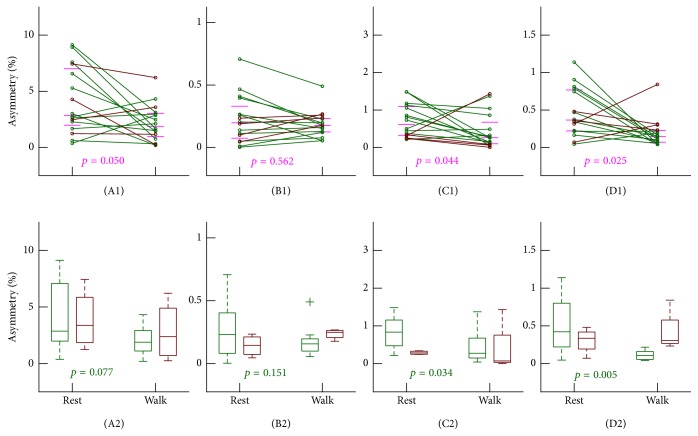
The asymmetries of short-term heart rate variability after wavelet detrending. In order to show the changes, results from the same individual were connected by lines. Horizontal bars indicate the median and [1st, 3rd] quartiles. *p* values were from Wilcoxon signed-rank test of each pair. ((A1) and (A2)) ΔPI; ((B1) and (B2)) ΔGI; ((C1) and (C2)) ΔSI; ((D1) and (D2)) ΔAI.* Rest*: results under resting seated position;* Walk*: results during regular walking. 13 individuals show reduction from* Rest* to* Walk* in (A1), 12 individuals show reduction from* Rest* to* Walk* in (C1), and 12 individuals show reduction from* Rest* to* Walk* in (D1). Results from males and from females are marked in different colors (male: green; female: brown). Lower panels summarize the box plots (that show from top to bottom the max, 3rd quartile, median, 1st quartile, and min) for males and females, separately. Outliers, if there are any, are marked by “+.”

Wavelet detrending did not change the HRA levels significantly under resting seated position (all* p*'s > 0.05 versus results from raw HRV data as revealed by the Wilcoxon signed-rank test). Similarly, the HRA levels during walking did not show significant changes after wavelet detrending (all* p*'s > 0.1 for PI, GI, and SI) except that assessed by AI which indicated a significant reduction (*p* = 0.04; 11 out of 16 subjects showed reduced AI after wavelet detrending). As a consequence, AI indicated significantly lower HRA during walking than that under resting seated position (*p* = 0.025;* d* = 0.7; 12 out of 16 subjects showed reduction; Figure 4(D1)). SI also indicated significantly lower HRA during walking (*p* = 0.044;* d* = 0.4; 12 out of 16 subjects showed reduction; Figure 4(C1)). The remaining two metrics showed consistent results as compared with those based on raw HRV data; that is, PI reduced significantly (*p* = 0.050;* d* = 0.6; 13 out of 16 subjects showed reduction) while GI showed no significant changes (*p* = 0.562; Figures 4(A1) and 4(B1)). Within the three metrics that showed statistical significance (i.e., PI, SI, and AI), the four female subjects did not display consistent changing patterns (i.e., for each metric there are both decrease and increase during walking across the four female subjects). The between-condition changes remain when restricting data to male subjects only (Figures 4(A2)–4(D2)), except that the reduction during walking in PI becomes borderline significant (*p* = 0.077; Figure 4(A2)).

## 4. Discussions

Asymmetry is an accepted intrinsic property of HRV. It imparts the time irreversibility of HRV—an important marker of the nonlinearity in HRV dynamics that can be perturbed by many pathologies [19]. For example, perturbed HRA has been observed in diseases including arrhythmia [21], heart failure [24], obstructive sleep apnea [25], myocardial infarction [26, 27], postoperative myocardial ischemia [28], and type 1 diabetes [29]. Most interestingly, HRA has suggested potential for postinfarction risk prediction [30]. The current pilot study explores whether and how HRA changes during regular walking. To answer the question, we used 5 min ECG data that applied a within-subject, randomized “crossover” design to examine changes of short-term HRV during exercise [15]. ECG data of each participant were monitored two times that correspond to a resting seated position and a regular walking protocol on the treadmill, respectively. We assessed the HRA using four established HRA metrics, that is, PI, GI, SI, and AI. With the 5 min ECG data, mainly the short-term HRA is expected to be captured [12, 19, 30] while the components related to long-term HRA may only have slight contributions to results, which limits the availability of long-term HRA to be examined fairly. Therefore, in this study we focused only on short-term HRA, and in order to further get rid of the potential weak contributions of long-term HRA, we repeated the calculations of the four HRA metrics on HRV recordings after a wavelet detrending process that removes the low-frequency components of <~0.03 Hz which contribute primarily to long-term HRA.

Our pilot data on 16 healthy college students showed a reduced PI while unchanged GI, SI, and AI during walking on treadmill based on raw HRV data. Based on wavelet-detrended data, reduced PI, SI, and AI were documented while GI still indicated no significant changes. It has been hypothesized that short-term HRA possesses a dominant contribution of vagal activity [9, 12]. Thus, the reduced PI observed from both raw HRV and detrended HRV data may underline the belief that vagal tone is withdrawn during low-intensity exercise [17, 31, 32]. However, none of the remaining three metrics, that is, GI, SI, and AI, showed significant changes based on raw HRV data, suggesting a possible lack of sensitivity to vagal withdrawal. Furthermore, SI and AI indicated significant decreases during walking using detrended HRV data, suggesting that, in addition to short-term HRA, SI and AI may also capture long-term HRA that confounds the changes of short-term HRA even though the contribution of long-term component in 5 min ECG data is low. GI was almost unchanged after detrending, implying that GI, a second-dimensional metric that relies on the distances, may capture mostly long-term HRA. We note that even with significant observations, the changing directions of these metrics with regular walking in several individuals are totally opposite (see Figures 3 and 4). Different changing directions may reflect different autonomic responses across individuals to the walking stimuli. The difference may come from different exercise habits, different levels of college study stress, or even autonomic disorders [33]. This information will be collected in our future studies in order to uncover what leads to the differences.

Consistently, all our results still held when using data of male subjects. However, with only four females, we could not reliably perform any statistical analyses. Besides, the changing directions of HRA from resting to walking conditions seemed not consistent. Together, they limited our ability to conclude anything for female subjects. In a previous study, an interesting sex difference in HRA in particularly younger subjects has been reported [34]. Further studies are thus warranted to examine whether the effect of regular walking on HRA differs across sexes. In addition, participants in the current study were all quite young. How age influences the effect of regular walking is yet another concern that requires further elucidations.

Our results also show consistency with some published work. For example, there are studies that observed decreased HRA during acute mental stress (i.e., Stroop and arithmetic test) [35] and aerobic exercise [36], both corresponding to an autonomic balance shift towards sympathetic predominance or vagal withdrawal. However, in the study that applied acute mental stress [35], GI was found to better reflect vagal withdrawal than PI did, which is different from what we observed. This difference may partially due to different data lengths used (i.e., 6 min in the mentioned study versus 5 min in ours). And another possible reason might be that we calculated the absolute difference of the actual HRA metrics and 50 (see Section 2.5, and more discussions regarding this can be found in the next paragraph). In a different study, the same group (i.e., the group of the mental stress study) also showed that HRA increased significantly during orthostasis and that GI was more sensitive to the stimulus [37]. HRV of ~15 min was used in that study. The increased HRA during orthostasis based on this relatively long data might reflect mainly the sympathetic activation, and the better performance of GI could thus be understandable as our results before and after detrending provide a hint that GI may be more sensitive to the sympathetic modulation and thus the long-term HRA. What is interesting is that the changing directions of short- and long-term HRA during vagal withdrawal or sympathetic activation are completely opposite which is worth further elucidations. An increase in HRA has also been observed with respiratory maneuver (e.g., inspiration/expiration = 2 : 1 or 1 : 1) [38]. Three 4.5 s metronome breathing patterns (1 : 1, 2 : 1, and normal pattern ~1 : 2) were administered for each participant while ECGs were recorded for 5 min at each breathing pattern. However, in that study no significant differences in traditional HRV parameters (such as power of higher frequency—the marker for vagal activity) were found. Further examinations to clearly figure out what led to the observed HRA changes are thus still required and this also limits the direct comparison between our study and the other three studies reviewed above, that is, [35–37], which attribute HRA changes mainly to autonomic responses.

It is worth noting that we used the absolute difference of an HRA metric to 50 as the index of asymmetric level (see Section 2.5). By calculating the absolute difference, we lost the power to differentiate the contributions of instantaneously accelerated and decelerated patterns. However, in the currently study, we focused mainly on the “asymmetry” phenomenon, which is believed to be existing especially during resting state as reported by many previous studies [3, 12], instead of the unbalanced sympathetic or vagal tones. The calculation of absolute changes provides the possibility of screening more asymmetric patterns out, as stated by a previous study [39]. In addition, the regulation of heart rate is not instantaneous. Instead, it takes a couple of seconds [40], which imparts the importance of measuring the symmetry of changes rather than the exact acceleration or deceleration patterns [39].

Our pilot study also touches a potential important point in short-term HRA analysis—the influence of nonstationary trend. To the best of our knowledge, this has not been considered seriously in previous work. We note that the very low-frequency component of <~0.03 Hz is usually considered nonstationary trend and its removal will hardly affect the beat-to-beat decelerating/accelerating patterns. However, it may affect how much the pattern deviates from symmetric. Therefore, if an HRA algorithm takes the position of the patterns in the Poincaré plot (either above or below the line of identity) into consideration, the results after trend removal would rarely get affected (such as the case for the metric PI; see Figures 3 and 4). If an algorithm considers the distance or area characteristics, it is possible that a pattern will be considered deviated a little bit more from symmetric before trend removal than from afterwards. This effect will be important when considering long-term asymmetry. In this scenario, a decreased asymmetry (increased symmetry) for the same recording after trend removal would thus be expected when using metrics such as SI and AI, especially for data during walking (which are true when comparing Figures 3 and 4). This also provides a possible explanation that PI decreases significantly during exercise both before and after trend removal while significant changes in SI and AI are only observed after trend removal. Based on our pilot data, nonstationary trend removal is recommended for short-term HRA analysis, and to validate this, definitely further examinations with more participants and different stimulus are warranted.

Recent advances in smart wearables open a new avenue for the monitoring and management of individual's health during daily routine. Perhaps one of the most common wearable devices is the ECG or heart rate monitor that can be used to assess the cardiovascular function and the underlying autonomic control status. A simplest idea in using such devices is to implement the algorithms that are previously developed based on data episodes collected during one time clinic or laboratory visit into continuous data, which provides the opportunity for sporadic, health-related alterations to be picked up, as well as the feasibility to look at the variations of these markers with time throughout a day.

## References

[1] Hon E. H., Lee S. T. (1965). Electronic evaluations of the fetal heart rate patterns preceding fetal death, further observations.

[2] Task Force of the European Society of Cardiology (1996). Heart rate variability, standards of measurement, physiological interpretation, and clinical use.

[3] Costa M., Goldberger A. L., Peng C.-K. (2005). Broken asymmetry of the human heartbeat: loss of time irreversibility in aging and disease.

[4] Costa M. D., Peng C.-K., Goldberger A. L. (2008). Multiscale analysis of heart rate dynamics: entropy and time irreversibility measures.

[5] Casali K. R., Casali A. G., Montano N. (2008). Multiple testing strategy for the detection of temporal irreversibility in stationary time series.

[6] Cammarota C., Rogora E. (2007). Time reversal, symbolic series and irreversibility of human heartbeat.

[7] Hou F., Zhuang J., Bian C. (2010). Analysis of heartbeat asymmetry based on multi-scale time irreversibility test.

[8] Acharya U. R., Joseph K. P., Kannathal N., Lim C. M., Suri J. S. (2006). Heart rate variability: a review.

[9] Piskorski J., Guzik P. (2012). Compensatory properties of heart rate asymmetry.

[10] Hainsworth R., Malik M. (1998). Physiology of the Cardiac Autonomic System.

[11] Nicolini P., Ciulla M. M., Asmundis C. D., Magrini F., Brugada P. (2012). The Prognostic Value of Heart Rate Variability in the Elderly, Changing the Perspective: From Sympathovagal Balance to Chaos Theory.

[12] Piskorski J., Guzik P. (2011). Asymmetric properties of long-term and total heart rate variability.

[13] Akintola A. A., van de Pol V., Bimmel D., Maan A. C., van Heemst D. (2016). Comparative analysis of the equivital EQ02 lifemonitor with holter ambulatory ECG device for continuous measurement of ECG, heart rate, and heart rate variability: A validation study for precision and accuracy.

[14] Kristiansen J., Korshøj M., Skotte J. H. (2011). Comparison of two systems for long-term heart rate variability monitoring in free-living conditions—a pilot study.

[15] Shi B., Zhang Y., Yuan C., Wang S., Li P. (2017). Entropy Analysis of Short-Term Heartbeat Interval Time Series during Regular Walking.

[16] Karmakar C., Khandoker A., Palaniswami M. (2012). Investigating the changes in heart rate asymmetry (HRA) with perturbation of parasympathetic nervous system.

[17] Boettger S., Puta C., Yeragani V. K. (2010). Heart rate variability, QT variability, and electrodermal activity during exercise.

[18] Li P., Liu C., Zhang M., Che W., Li J. (2011). A Real-Time QRS Complex Detection Method.

[19] Porta A., Casali K. R., Casali A. G. (2008). Temporal asymmetries of short-term heart period variability are linked to autonomic regulation.

[20] Guzik P., Piskorski J., Krauze T., Wykretowicz A., Wysocki H. (2006). Heart rate asymmetry by Poincaré plots of RR intervals.

[21] Karmakar C. K., Khandoker A. H., Palaniswami M. (2015). Phase asymmetry of heart rate variability signal.

[22] Yan C., Li P., Ji L., Yao L., Karmakar C., Liu C. (2017). Area asymmetry of heart rate variability signal.

[23] Sawilowsky S. S. (2009). Very large and huge effect sizes.

[24] Woo M. A., Stevenson W. G., Moser D. K., Trelease R. B., Harper R. M. (1992). Patterns of beat-to-beat heart rate variability in advanced heart failure.

[25] Guzik P., Piskorski J., Awan K., Krauze T., Fitzpatrick M., Baranchuk A. (2013). Obstructive sleep apnea and heart rate asymmetry microstructure during sleep.

[26] Huikuri H. V., Seppänen T., Koistinen M. J. (1996). Abnormalities in beat-to-beat dynamics of heart rate before the spontaneous onset of life-threatening ventricular tachyarrhythmias in patients with prior myocardial infarction.

[27] Stein P. K., Domitrovich P. P., Huikuri H. V., Kleiger R. E. (2005). Traditional and nonlinear heart rate variability are each independently associated with mortality after myocardial infarction.

[28] Laitio T. T., Mäkikallio T. H., Huikuri H. V. (2002). Relation of heart rate dynamics to the occurrence of myocardial ischemia after coronary artery bypass grafting.

[29] Guzik P. (2005). Heart rate variability by Poincaré plot and spectral analysis in young healthy subjects and patients with type 1 diabetes.

[30] Guzik P., Piskorski J., Barthel P. (2012). Heart rate deceleration runs for postinfarction risk prediction.

[31] White D. W., Raven P. B. (2014). Autonomic neural control of heart rate during dynamic exercise: revisited.

[32] Fisher J. P. (2014). Autonomic control of the heart during exercise in humans: Role of skeletal muscle afferents.

[33] Fu Q., Levine B. D. (2013). Exercise and the autonomic nervous system.

[34] Voss A., Schroeder R., Heitmann A., Peters A., Perz S. (2015). Short-term heart rate variability - Influence of gender and age in healthy subjects.

[35] Visnovcova Z., Mestanik M., Javorka M. (2014). Complexity and time asymmetry of heart rate variability are altered in acute mental stress.

[36] Torres B. D. L. C., Orellana J. N. (2010). Multiscale time irreversibility of heartbeat at rest and during aerobic exercise.

[37] Chladekova L., Czippelova B., Turianikova Z. (2012). Multiscale time irreversibility of heart rate and blood pressure variability during orthostasis.

[38] Klintworth A., Ajtay Z., Paljunite A., Szabados S., Hejjel L. (2012). Heart rate asymmetry follows the inspiration/expiration ratio in healthy volunteers.

[39] Khandoker A. H., Karmakar C., Brennan M., Palaniswami M., Voss A. (2013).

[40] Eckberg D. L. (1980). Nonlinearities of the human carotid baroreceptor-cardiac reflex.

